# Current approaches to develop “off-the-shelf” chimeric antigen receptor (CAR)-T cells for cancer treatment: a systematic review

**DOI:** 10.1186/s40164-023-00435-w

**Published:** 2023-08-21

**Authors:** Cristina Aparicio, Carlos Acebal, Margarita González-Vallinas

**Affiliations:** 1 Unit of Excellence Institute of Biomedicine and Molecular Genetics of Valladolid (IBGM), Universidad de Valladolid (UVa)-CSIC, Valladolid, Spain; 2 Department of Biochemistry, Molecular Biology and Physiology, Faculty of Medicine, Universidad de Valladolid, Valladolid, Spain

**Keywords:** Chimeric antigen receptor (CAR)-T cells, Cancer immunotherapy, “Off-the-shelf” adoptive T cell therapy, Allogeneic treatment, Advanced therapy medicinal products (ATMPs), Genetic engineering, Graft-versus-host disease (GvHD), Allorejection, Systematic review

## Abstract

Chimeric antigen receptor (CAR)-T cell therapy is one of the most promising advances in cancer treatment. It is based on genetically modified T cells to express a CAR, which enables the recognition of the specific tumour antigen of interest. To date, CAR-T cell therapies approved for commercialisation are designed to treat haematological malignancies, showing impressive clinical efficacy in patients with relapsed or refractory advanced-stage tumours. However, since they all use the patient´s own T cells as starting material (i.e. autologous use), they have important limitations, including manufacturing delays, high production costs, difficulties in standardising the preparation process, and production failures due to patient T cell dysfunction. Therefore, many efforts are currently being devoted to contribute to the development of safe and effective therapies for allogeneic use, which should be designed to overcome the most important risks they entail: immune rejection and graft-versus-host disease (GvHD). This systematic review brings together the wide range of different approaches that have been studied to achieve the production of allogeneic CAR-T cell therapies and discuss the advantages and disadvantages of every strategy. The methods were classified in two major categories: those involving extra genetic modifications, in addition to CAR integration, and those relying on the selection of alternative cell sources/subpopulations for allogeneic CAR-T cell production (i.e. γδ T cells, induced pluripotent stem cells (iPSCs), umbilical cord blood T cells, memory T cells subpopulations, virus-specific T cells and cytokine-induced killer cells). We have observed that, although genetic modification of T cells is the most widely used approach, new approaches combining both methods have emerged. However, more preclinical and clinical research is needed to determine the most appropriate strategy to bring this promising antitumour therapy to the clinical setting.

## Background

**Fig. 1:**
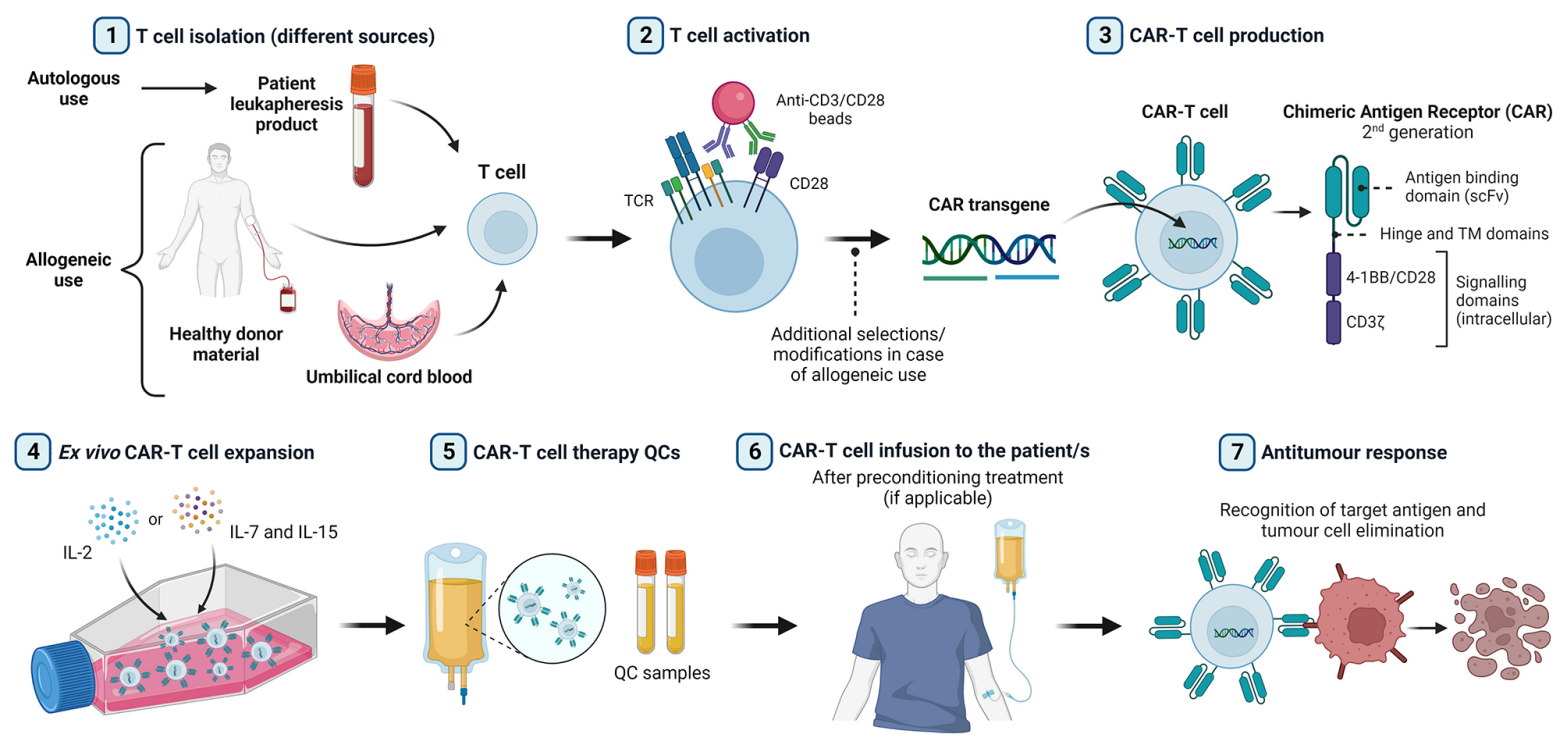
Overview of general autologous/allogeneic CAR-T cell production and application process. **(1)** T cell isolation from different sources (depending on autologous or allogeneic use); **(2)** T cell activation for *ex vivo* culture (usually through CD3 and CD28 stimulation); **(3)** genetic modification for inducing CAR expression (commonly 2^nd^ generation CARs); **(4)** expansion to obtain the desired number of cells (usually either IL-2 or IL-7 and IL-15 supplementations); **(5)** CAR-T cell therapy quality controls (in-process and final product controls); **(6)** administration to the patient/s; and **(7)** elimination of tumour cells triggered by CAR antigen recognition. QC: quality control. TM: transmembrane. Created with BioRender.com.

Chimeric antigen receptor (CAR)-T cell therapy has arisen as one of the most promising therapeutic strategies in cancer treatment. It is a class of advanced therapy medicinal product (ATMP) based on the use of T cells that are genetically modified to express a CAR, which directs their activity against tumour cells expressing the antigen of interest [1]. CAR molecules consist mainly of: (i) an extracellular target antigen-binding domain, which is commonly an antibody-derived single-chain variable fragment (scFv), (ii) a hinge region, (iii) a transmembrane domain, and (iv) an intracellular signalling domain that mediates T cell activation (CD3ζ). Second-generation CARs incorporate a co-stimulatory intracellular domain (usually CD28 or 4-1BB), and third-generation CARs include two co-stimulatory domains [2]. Furthermore, fourth generation CAR-T cells contain additional features that modulate their efficacy such as expression of secreting molecules (cytokines, T cell engagers, agonists or inhibitors of different cell receptors, etc.) or membrane receptors (e.g. chemokine receptors) [3]. The general manufacturing procedure and application of CAR-T cell therapy is illustrated in Fig. 1.

## Landscape of approved CAR-T cell therapies

To date, all CAR-T cell therapies approved for commercialisation by the Food and Drug Administration (FDA) and the European Medicines Agency (EMA) are directed to treat relapsed or refractory (r/r) haematological malignancies. The first CAR-T therapy, tisagenlecleucel (Kymriah™) was approved by the FDA in 2017 to treat r/r acute lymphoblastic leukaemia (ALL) and the following year it was authorised against r/r diffuse large B cell lymphoma (DLBCL) [4]. In Europe, it was authorised by the EMA for both indications in 2018 [5]. Axicabtagene ciloleucel (Yescarta™) was also commercialised in 2017 for certain types of r/r large B cell lymphoma (LBCL) such as DLBCL, primary mediastinal large B cell lymphoma, high grade B cell lymphoma or DLBCL secondary to follicular lymphoma, and later (2021) also for follicular lymphoma [6]. Brexucabtagene autoleucel (Tecartus™) was FDA-approved in 2020 for r/r mantle cell lymphoma (MCL) and, in 2021, for r/r B cell precursor ALL [7]. In 2021, lisocabtagene maraleucel (Breyanzi™) was authorised for r/r LBCL including DLBCL, high grade B cell lymphoma, primary mediastinal large B cell lymphoma, and follicular lymphoma grade 3B. Idecabtagene vicleucel (Abecma™) was commercialised in the same year for r/r multiple myeloma [8, 9]. The last FDA- and EMA-approved CAR-T cell product has been ciltacabtagene autoleucel (Carvykti™) in 2022, for multiple myeloma [10]. In addition, in 2021, the Spanish Agency of Medicines and Medical Devices (AEMPS) approved for ALL, with a special local authorisation called “hospital exemption”, the product ARI-0001, a non-industrially manufactured CAR-T cell therapy developed at the Hospital Clínic of Barcelona that is the first approved medicine of this kind developed entirely in Europe [11].

Regarding the antigens of the commercialised antitumour CAR-T therapies, idecabtagene vicleucel and ciltacabtagene autoleucel are directed against the B cell maturation antigen (BCMA), which overexpression and activation is associated to multiple myeloma, while all the others are designed to target CD19, a marker of B cells. Moreover, all of them are for autologous use, which implies that the patient´s own T cells are the starting material for manufacturing. Therefore, these therapies must be produced in a personalised manner, thus entailing a number of disadvantages that mainly limit their clinical application (developed in a subsequent section). With regard to CAR generation, the commercialised products are based on second-generation CARs, using CD28 or 4-1BB as co-stimulatory domain fused to CD3ζ signalling domain for full T cell activation [4, 6–10]. Lisocabtagene maraleucel additionally expresses a non-functional truncated epidermal growth factor receptor (EGFRt), which induces the ablation of the CAR-T cells in vivo when an antibody against this receptor (e.g. cetuximab) is administered [8, 12].

In addition, CAR technology has been also applied to regulatory T cells (Tregs), which possess immunoregulatory function, in order to develop CAR-Tregs directed to promote tolerance to skin allografts [13] or pancreas (in cases of organ transplant or type 1 diabetes) [14, 15] and to treat graft-versus-host disease (GvHD) [16].

## Therapeutic efficacy and main complications of CAR-T cells

CAR-T cell therapy has emerged as a strategic need for cancer immunotherapy to treat haematological tumours, which are, in general, very aggressive diseases, thus resulting in poor patient outcomes. For example, ALL has a very bad prognosis in adult population with a long-term (5 years) survival rate of 35–45% [17]. Conversely, with the same chemotherapy strategy, paediatric population is highly curable with a long-term survival rate of 90% [17, 18]; however, this decreases to 50% after the first tumour relapse [18]. Regarding DLBCL, 60% of patients achieve an effective remission after rituximab and multiagent chemotherapy treatment. Nevertheless, about 30–40% of patients relapse and 10% are refractory primary cases [19].

In contrast, according to a meta-analysis evaluating the efficacy of CAR-T therapies axicabtagene ciloleucel, tisagenlecleucel and lysocabtagene maraleucel in different haematological tumours, the complete response rate is over 50% and the complete and/or partial response rate is nearly 70% [20]. For this reason, CAR-T cell therapy has experienced a great growth in recent years; however, it is generally being indicated only for patients with r/r disease after at least two lines of treatment.

Regarding the real-world data, the results obtained in comparison with the pivotal clinical trials are very similar. For example, the overall response rate (ORR) and the complete response rate (CRR) for tisagenlecleucel in r/r DLBCL were 66% and 42%, respectively, in the real-world data, and 52% and 40% in the pivotal clinical trial (JULIET). In the case of axicabtagene ciloleucel in r/r DLBCL, the ORR and CRR were 80% and 60%, respectively, and 82% and 58% in the pivotal clinical trial (ZUMA-1). In this study, they showed that the ORR/CRR was better with axicabtagene ciloleucel and also the 1-year progression-free survival (46.6% axicabtagene ciloleucel and 33.2% tisagenlecleucel) and the 1-year overall survival (63.5% and 48.8%, respectively) [21].

The main complications of CAR-T cell therapy are the cytokine release syndrome (CRS), which is characterised by a systemic inflammatory response derived from an exacerbated immune response, and the immune effector cell-associated neurotoxicity syndrome (ICANS), which is a non-site-specific central nervous system involvement [22]. According to the meta-analysis of axicabtagene ciloleucel, tisagenlecleucel and lysocabtagene maraleucel, 13% of all treated patients had severe CRS and 22% developed ICANS [20]. In a comparative analysis between axicabtagene ciloleucel and tisagenlecleucel with real-world data in r/r DLBCL patients, the incidence of grade 1–2 CRS was greater in axicabtagene ciloleucel but that of grade 3 CRS was not significantly different. Regarding ICANS, it was significantly more frequent (both grades 1–2 and ≥ 3) after the treatment with axicabtagene ciloleucel [21].

An additional important issue regarding CAR-T cell therapy is target antigen selection and CAR design, which greatly define the specificity, efficacy and safety of the treatment. The identification of non-malignant tissues expressing the CAR target and the consequent possible on-target off-tumour effects is critical before patient application. Currently, this is one of the most relevant problems to be solved in the designed of novel CAR-T cell therapies, especially to treat solid tumours [23].

## Limitations of autologous CAR-T cell therapies and the potential of allogeneic use

The commercialised autologous CAR-T cell therapies are achieving great clinical results, with complete responses even in patients with advanced tumours not responding to conventional treatments; however, patients’ T cells may be dysfunctional and exhausted, which influences the potency and variability of CAR-T cell products. This can be caused by patient age, the number of previous lines of treatment, the disease itself and, in solid tumours, also by local immune suppression and the effects of prolonged T cell stimulation [24, 25]. In addition, autologous CAR-T cell therapies are individualised products, thus entailing theoretically higher costs and manufacturing time, usually around 2–3 weeks [26, 27]. Moreover, in patients with refractory leukaemias, there are often large numbers of circulating leukaemic cells that can be extracted along with healthy lymphocytes and thus contaminate the product. It has been suggested that CAR-transduced cancer cells present on therapy may be associated with down-regulation of the target antigen leading to patient relapse by this newly generated population [28].

The production of CAR-T cells for allogeneic use may overcome the drawbacks of autologous therapies. The employment of healthy donor T cells can increase the viability and accessibility of the treatment. Moreover, it allows the availability of cryopreserved batches for immediate treatment, greater standardisation of the product, and the possibility of re-dosing and combining CAR-T cells directed against different targets [26, 27]. Interestingly, recent studies focus on reducing the time for CAR-T cell manufacturing, and an autologous anti-CD19 CAR-T cell therapy has been obtained in less than two days that, in comparison with common longer production processes used to manufacture commercialised CAR-T therapies, show increased proportion of naïve T cells (Tn) / stem cell memory T cells (Tscm), higher antitumour potency and cytokine secretion in vitro and enhanced expansion and antitumour efficacy in vivo [29]. The CAR-T cell product manufactured with the novel process is being used in a clinical trial that is so far demonstrating a positive safety profile [30]. However, patient-derived CAR-T cells have shown reduced potency compared to healthy donor-derived CAR-T cells and even among healthy donors there are differences in proliferation and cytotoxicity that appear to be related to the proportion of memory T cell subtypes at the beginning of the manufacturing [25]. Therefore, the use of donor-derived T cells and donor selection for CAR-T cell manufacturing would largely contribute to product standardisation. Table 1 summarises the advantages and disadvantages of autologous and allogeneic CAR-T cells.

**Table 1:** Autologous vs. allogeneic CAR-T cell therapy: advantages and disadvantages. (GvHD: graft-versus-host disease)

	AUTOLOGOUS CAR-T CELLS	ALLOGENEIC CAR-T CELLS	
**ADVANTAGES**	No risk of GvHD	Potential GvHD	**DISADVANTAGES**
	Intermediate to long persistence (months or years). No immune rejection.	Limited in vivo persistence: need of additional modifications or patient immunosuppression. Potential immune rejection.	
**DISADVANTAGES**	High costs of manufacturing	Possibility of large-scale manufacturing and, consequently, important potential cost reduction	**ADVANTAGES**
	Time of manufacturing (typically > 1 week) may cause delays in patient treatment	Immediate availability for patient treatment	
	Donor-dependent manufacturing efficiency	Possibility of product standardisation	
	Characteristics of T cells affected by patient age, cancer disease and/or treatments	Optimal quality of T cells due to obtaining from healthy donors (viability, proliferation and potency) and possibility of donor selection	
	Risk of product contaminated with tumour cell (leukaemias; rare but serious)	No risk of product contamination by tumour cells	

To develop a safe allogeneic therapy, two major potential problems with the allogeneic use of T cells must be resolved: GvHD, which is mainly produced by a T cell receptor (TCR)-mediated immune response to host tissues that can be life-threatening, and graft rejection by the recipient’s immune system, which can limit the effectiveness of the therapy [26, 31]. This is because the host immune cells, mainly by detecting foreign human leukocyte antigen (HLA) class I and class II molecules, recognise allogeneic cells and eliminate them [32]. In contrast, autologous therapy does not cause GvHD and, additionally, it does not suffer immune rejection, therefore persisting longer in vivo [26, 31].

The rapid advances in the development of CAR-T cell therapies in recent years and the promising potential of CAR-T cells for allogeneic use raises the need for a comprehensive review of the wide range of different production strategies for allogeneic CAR-T cell therapies that try to avoid the appearance of the problems mentioned above. To our knowledge, the different strategies for developing “off-the-shelf” CAR-T cells, ranging from genetic engineering to selection of specific T cell subpopulations, have not been systematically reviewed before.

## Materials and methods

This systematic review was performed in compliance with the Preferred Reporting Items for Systematic Reviews and Meta-Analyses (PRISMA) guidelines [33].

### Search strategy

We tried to identify all publications describing strategies for the production of allogeneic CAR-T cell therapies. To that end, PubMed database was systematically searched, in October 2022, using the following combination of terms: “(chimeric antigen receptor[Title/Abstract] OR CAR[Title/Abstract]) AND (T cell[Title/Abstract]) AND (allogeneic[Title/Abstract] OR donor derived[Title/Abstract] OR virus specific[Title/Abstract] OR off the shelf[Title/Abstract])”. Additionally, in order to exclude reviews, meta-analysis, articles related to allogeneic hematopoietic stem cell transplantation and CAR-modified cells different than T cells, we added in the search the following: “NOT (review[Publication Type]) NOT (systematic review[Publication Type]) NOT (meta-analysis[Publication Type]) NOT (cell, NK[MeSH Terms]) NOT (hematopoietic stem cell transplantation[MeSH Terms]) NOT (stem cell transplant[Title/Abstract]) NOT (stem cell transplantation[Title/Abstract]) NOT (CAR NK[Title/Abstract]) NOT (chimeric antigen receptor NK[Title/Abstract]) NOT (CAR-macrophage cells[Title/Abstract])”.

### Eligibility criteria

The reported data was screened with the following inclusion criteria: investigations that studied strategies for the production of allogeneic CAR-T cell therapies. The exclusion criteria were as follows: studies not using/describing (1) an allogeneic therapy strategy, (2) a CAR therapy or (3) a therapy based on T cells; (4) studies related to CAR therapy targeting viral diseases; (5) studies with CAR-Tregs, not intended for cancer treatment; (6) studies focusing only on CAR-T manufacturing systems; (7) reviews; (8) meta-analysis; (9) news; (10) interviews; and (11) studies not available in full text in English or Spanish languages.

### Study selection

Obtained search results were screened based on title and abstract. In a second step, the full texts of the remaining papers were evaluated to be included in the systematic review. Study screening and selection was performed by two independent reviewers.

## Results

### Selection of relevant studies

**Fig. 2:**
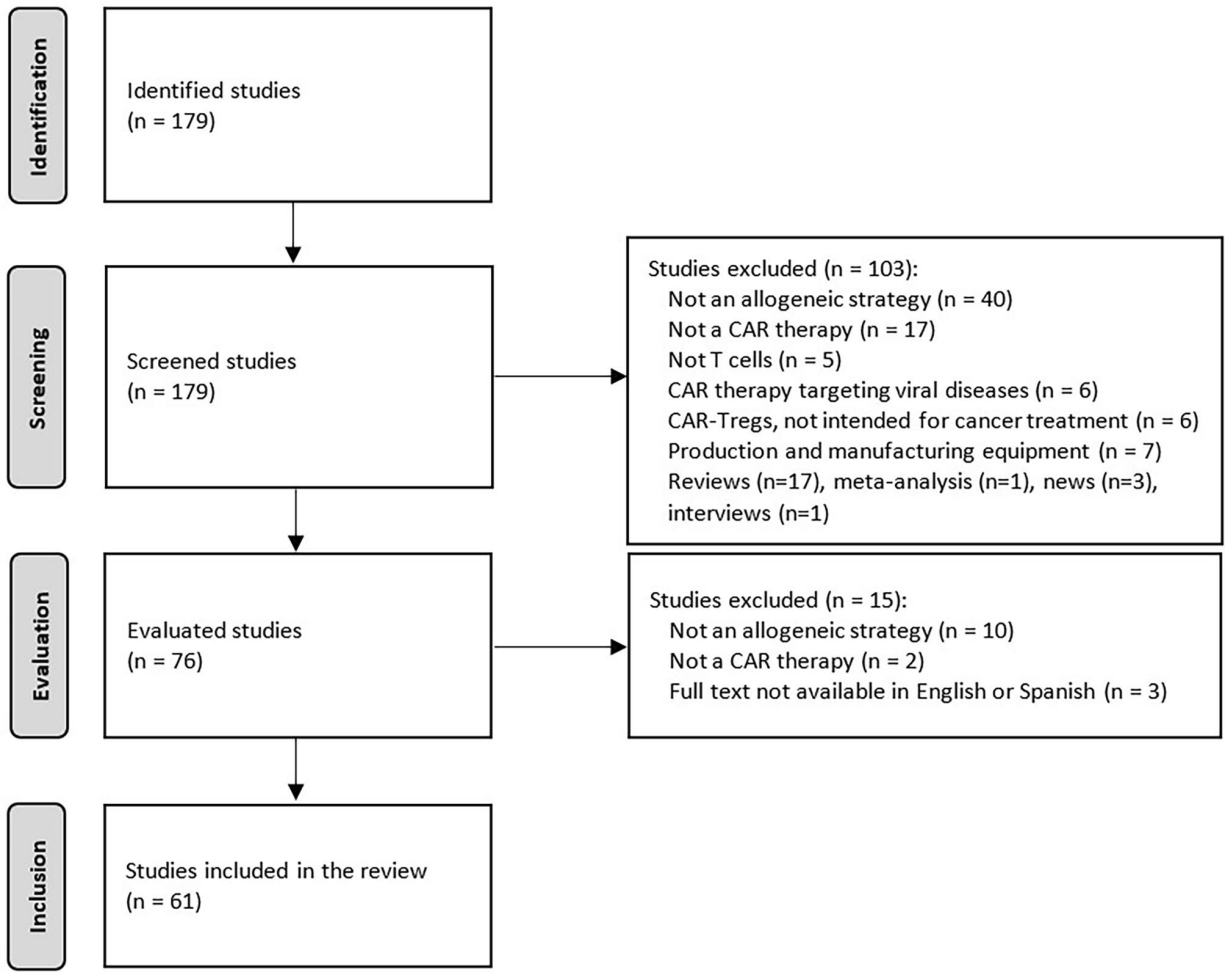
Flow diagram summarizing the selection process for studies included in the systematic review

**Fig. 3:**
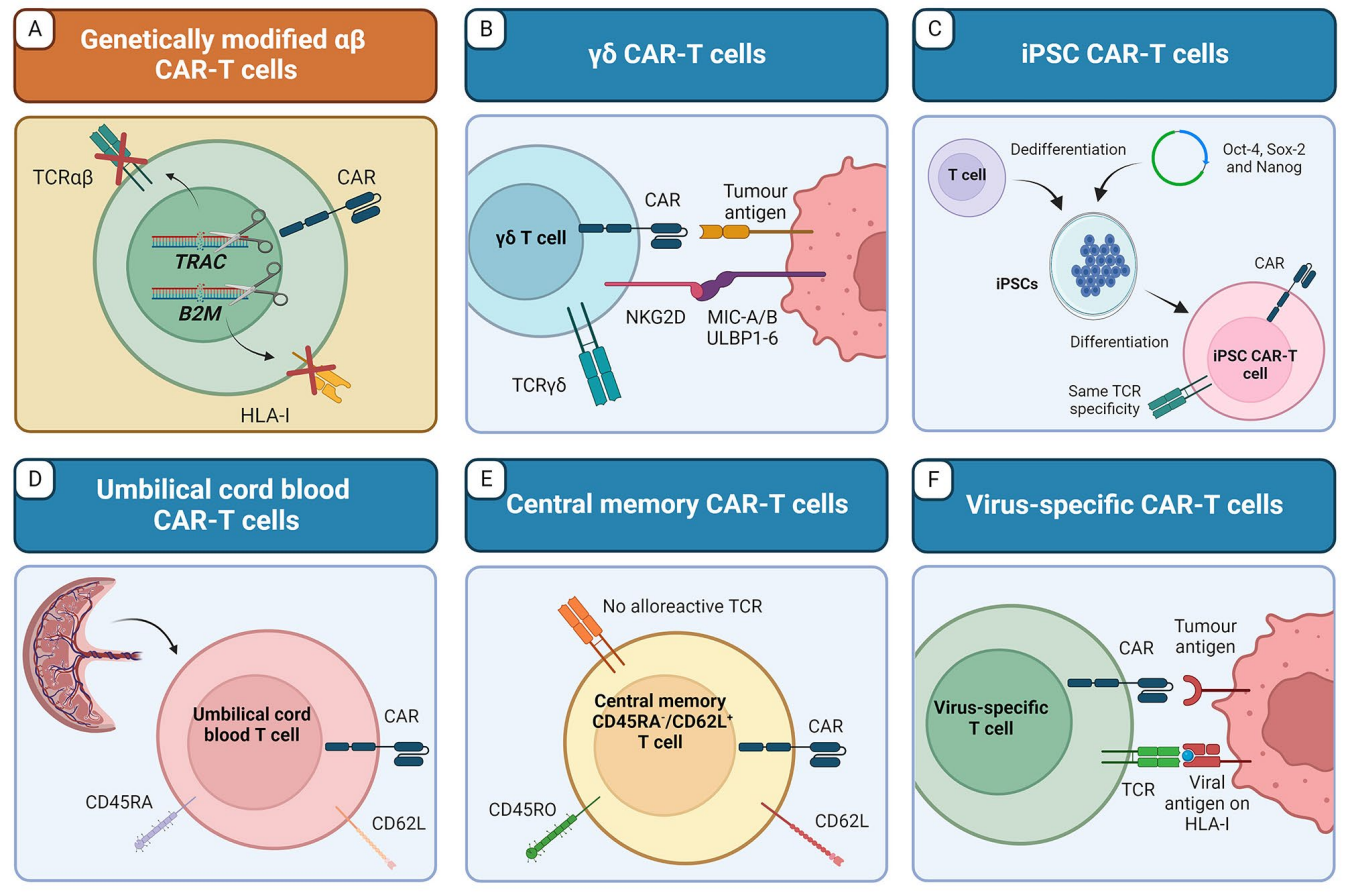
Representative examples of the main strategies to produce antitumour allogeneic CAR-T cells: (**A**) Performing additional genetic modifications (besides CAR transgene introduction) on donor αβ T cells, or using different cell sources such as (**B**) γδ T cells, (**C**) induced pluripotent stem cells (iPSCs), (**D**) umbilical cord blood T cells, (**E**) central memory T cells, or (**F**) virus-specific T cells. Created with BioRender.com.

The selection process for the systematic review is shown in the PRISMA flow diagram (Fig. 2). A total of 179 articles were initially identified using the search terms (see previous section) and 61 studies were finally included after screening and eligibility assessment. These articles were comprehensively read to identify the different strategies reported for allogeneic CAR-T cells production. We identified two main categories into which these strategies could be divided: (i) those involving extra genetic modifications, in addition to CAR integration, and (ii) those relying on the selection of alternative sources or subpopulations of T cells. The concrete strategies for allogeneic CAR-T cell production identified in the systematic review and their advantages and disadvantages are summarised in Fig. 3 and Table 2, respectively. In the following sections, we describe in detail the different strategies and the reported studies related.

**Table 2:** Advantages and disadvantages of different types of CAR-T cell therapy for allogeneic use (GvHD: graft-versus-host disease; iPSC: induced pluripotent stem cell; CIK: cytokine-induced killer)

STRATEGY	ADVANTAGES	DISADVANTAGES	REFERENCE/S
Genetically modified αβ CAR-T cells	• Designed to be non-immunogenic • High availability	• Additional steps of in vitro genetic manipulation • Risk of off-target mutations • Risk of GvHD due to residual TCRαβ^+^ T cells • Low proliferation *ex vivo* • High cost	[24, 25, 34–61]
γδ CAR-T cells	• HLA-independent antigen recognition (low risk of GvHD) • NK receptor expression • Cytotoxic phenotype • Efficacy in solid tumours	• Short in vivo persistence • Low availability • Difficult *ex vivo* proliferation	[62–70]
iPSC CAR-T cells	• Unlimited source • Homogeneous product (defined TCR specificity and HLA haplotype)	• Technically complex • High cost • Need of additional genetic modifications • Additional quality controls to avoid undifferentiated iPSCs, which would compromise safety	[71–77]
Umbilical cord blood CAR-T cells	• Low risk of GvHD • High proliferation capacity	• Limited number of cells	[78–80]
Central memory CAR-T cells	• Easy to obtain • Low prone to develop GvHD (non-alloreactive TCR) • Longer in vivo persistence than more differentiated T cells	• High percentage of effector memory T cells in the final product: lower in vivo persistence	[81–83]
Virus-specific CAR-T cells	• Viral TCR specificity (non-alloreactive TCR) • Additional protection against viral infections • Efficacy against virus-induced cancers • Possibility of boosting in vivo via TCR stimulation	• Short in vivo persistence (but increases upon TCR stimulation with viral antigens)	[84–91]
CIK CAR-T cells	• Highly cytotoxic phenotype (NK-like) • Minimal alloreactivity	• High cell heterogeneity • Low proportion of early memory T cells: limited in vivo persistence	[92]

### Additional genetic modifications for allogeneic CAR-T cell production

All CAR-T cells, both autologous and allogeneic, are genetically-modified T cells to express the specific CAR molecule. Moreover, one of the main strategies to enable the allogeneic use of CAR-T cells manufactured from healthy donor T cells involves the addition of extra genetic modifications in the manufacturing process.

#### Genetic strategies to enable allogeneic use of CAR-T cells

As previously mentioned, the two main potential problems of the allogeneic use of T cells are GvHD and immune rejection. The former can be avoided by eliminating the TCR, usually through the knockout of the constant domain of one of its chains (α or β), or by replacing some TCR subunits that impedes its antigen recognition function [45, 93]. However, although the absence of TCR controlled the tumour burden in vivo without alloreactivity, the long-term persistence was lower compared to CAR-T cells with endogenous TCR in patient-derived mouse xenografts [94].

Regarding immune rejection, it is avoided by preventing the expression of HLA class I (HLA-I) molecules by knocking out their common subunit β2-microglobulin (encoded by *B2M* gene), which prevents the recipient’s T cells from recognising the therapeutic cells as foreign through their TCR [36]. However, HLA-I negative T cells are susceptible of being lysed by host natural killer (NK) cells; to avoid this, a study applied the strategy of inducing the constitutive expression of mutant B2M-HLA-E and B2M-HLA-G fusion proteins [95]. Furthermore, activated T cells also express HLA class II (HLA-II) molecules, whose incompatibility can trigger the activation of receptor alloreactive CD4^+^ T cells [36]. Therefore, some authors propose for allogeneic CAR-T cell manufacturing knocking out either the *CIITA* gene, which is an important transcriptional activator of HLA-II genes [39], or the *HLA-DRA*, *-DQA* and -*DPA* genes, which encode the α-chains of HLA-II molecules, which are less polymorphic than β chains, thus resulting in higher efficiency for eliminating HLA-II expression [36].

Different gene editing technologies have been utilised for deleting the aforementioned genes, such as the CRISPR/Cas9 system [35], zinc finger nucleases (ZFNs) [58], or transcription activator-like effector nucleases (TALENs) [57]. CRISPR/Cas9 technology is the most widely used because it has demostrated a remarkably low rate of off-target mutagenesis in T cells [40, 48]. In addition, a specific high-fidelity Cas9 mutant, called eSpCas9, did not cause any detectable off-target effect, making it an even safer technology [48]. To study the expected decreased alloreactivity, Ren et al. co-cultured TCR^−^/HLA-I^−^ T cells and irradiated allogeneic peripheral blood mononuclear cells (PBMC) and observed that they triggered only a minimal response, which was hypothesised to be mediated by NK cells from the PBMC population [48]. Moreover, this group developed an allogeneic CAR-T cell prototype by introducing the gRNAs directed to eliminate TCR and HLA-I expression in a lentiviral vector together with the CAR transgene, thus achieving constitutive expression of the gRNAs and the consequent increase in the knockout efficiency and population homogeneity [35, 40].

Since multiplex editing with Cas9 nuclease may cause risk of gene rearrangements and chromosomal instability due to double-strand breaks, it has also been proposed the use of base-editing proteins, such as BE3 and BE4, aimed at inducing exon skipping by disrupting splice acceptor sites or creating premature stop codons, thus avoiding double-strand breaks and minimising the genetic risk [56].

Another technique employed to produce allogeneic CAR-T cells is TALEN, which has achieved only double gene disruption, in contrast to the CRISPR/Cas9 system by which quadruple knockouts have been reported [35]. This is owed to the complexity of the construct and the difficulty of targeting several genes with TALEN technology, which is also the case with ZFN [48]. Some groups have developed TALEN-edited CAR-T cells with different modifications such as TCR^−^/CD52^−^ [25, 41, 43] or TCR^−^/dCK^−^ [34], which avoid GvHD due to elimination of TCR and are resistant to other concomitant treatments such as alemtuzumab, a chemotherapeutic agent which targets CD52, or to purine nucleoside analogues, antitumour prodrugs activated by the enzyme deoxycytidine kinase (dCK), respectively [25, 34, 41, 43].

It has also been reported a strategy that combines TCR deletion with CAR transgene integration by using adeno-associated virus (AAV)-based vectors, in which the CAR is integrated in a targeted manner in the *TRAC* gene locus, thus avoiding the random integration of CAR gene that occurs with lentiviral vectors. Integration is achieved by homology-directed recombination after disrupting the *TRAC* gene by a nuclease such as Cas9 [24], *TRAC* megaTAL [52], or TRC1-2 (a single-chain variant of I-CreI) [53]. Jo et al. went a step further by targeting with TALEN the *TRAC* and *B2M* genes where they inserted the CAR and HLA-E encoding-genes, respectively, using AAVs. In this study, they observed an increase of persistence and antitumour effect of the allogeneic CAR-T cells in presence of NK cells in vivo and in vitro, respectively, due to HLA-E expression [60]. On the other hand, to avoid the use of viral vectors, Yang et al. used the same CAR targeted integration strategy using a TALEN nuclease and naked double-stranded DNA that included the CAR sequence [54].

In relation to immune rejection, K3 and K5, protein ubiquitin-ligases of human herpes virus-8, have been demonstrated to down-regulate together HLA class I (HLA-A, -B, -C) and HLA class II (HLA-DR), as well as the NK cell activating ligands MICA and MICB, thus avoiding both T cell and NK cell citotoxicity. This strategy would contribute to the production of allogeneic CAR-T cell therapies less prone to elimination by the recipient’s immune system [51]. Another challenge to be met, especially in the treatment of solid tumours, is the difficulty of trafficking and infiltration of the tumour and the immunosuppressive tumour microenvironment, together with the appearance of T cell exhaustion phenotype upon repeated activation [96]. In this regard, the inhibition of immune checkpoints using monoclonal antibodies is a widespread strategy in the field of oncology, given its good results in enhancing antitumour immunity. However, this approach entails the potential risk of breaking peripheral tolerance, which might trigger autoimmune responses [38]. Therefore, the silencing of genes encoding molecules involved in this signalling axis, including programmed cell death 1 (PD1) and cytotoxic T-lymphocyte antigen 4 (CTLA-4), which are inhibitory receptors related to T cell exhaustion and the immune escape of tumour cells, are being studied with the aim of increasing cytotoxic activity [24, 35, 38, 48]. Also, Ren et al. performed the knockout of Fas receptor to prevent the attenuation of CAR-T cell activity due to Fas-FasL cell signalling that triggers activation-induced cell death [35].

To increase the safety of these therapies, suicide switches can be inserted, such as inducible Caspase 9, which produces apoptosis upon administration of a small molecule drug [55], or rituximab-binding domains, which allow CAR-T cells to be depleted by administering this monoclonal antibody [25, 37, 42, 43].

In case of treating T cell malignancies, a major obstacle is that the CAR target antigen is shared by CAR-T cells and malignant T cells, which leads to therapeutic T cell fratricide. To avoid this, the target molecule should be eliminated in the therapeutic cells. Cooper et al. developed an “off-the-shelf” CAR-T cell therapy by eliminating TCR and CD7 expression in CAR-T cells directed towards CD7 antigen [47]. In this regard, GC027, a CAR-T cell product that shares these characteristics, has been studied in a phase I clinical trial with promising preliminary results [46].

Moreover, in order to avoid the residual TCR^+^ T cells that might cause GvHD, Juillerat et al. induced the transient expression of an additional CAR that targets CD3, which eliminate the TCR^+^ CAR-T cells, leading to a very high proportion of TCRαβ^−^ population in the final CAR-T cell product [44].

The strategies involving additional genetic modifications (besides introducing the CAR transgene) are very versatile and offer unlimited options not only to allow the allogeneic use of CAR-T cell products, but also to improve different characteristics of the therapeutic cells to increase persistence, infiltration, antitumour efficacy, safety profile, etc. Although high levels of efficiency can be achieved, for example, in gene knockout in human T cells (e.g. around 70–90% *TRAC* gene disruption obtained by using different procedures based on CRISPR/Cas9 technology [35]) and gene modification technologies are rapidly improving, these procedures need an important previous work to be optimised, and usually additional selection steps are needed during the manufacturing process to obtain the purified CAR-T cells with the desired phenotype.

#### Alternatives to TCRαβ elimination

Eliminating the membrane expression of TCR complex is the most widely used strategy for the production of allogeneic CAR-T cells based on additional genetic modifications. However, this involves the loss of cell surface expression of CD3 subunits, which are part of the TCR complex, and complicate their survival and expansion capacity *ex vivo*, as CD3/CD28 stimulation is often used for activation of T cells in culture [93].

An alternative approach was reported by Galetto et al. who replaced the expression of the TCRα subunit in T cells with a pre-TCRα, an endogenous substitute for the α-chain of the TCR involved in T cell maturation. To accomplish this, they eliminated the *TRAC* locus using TALEN and then inserted the gene encoding a truncated form of pre-TCRα by lentiviral transduction. The pre-TCRα is able to form disulphide bridges with the β-chains of the TCR and to form complexes with CD3 on the membrane, allowing subsequent stimulation by CD3 and thus cell proliferation. Moreover, they observed no evidence of GvHD when they infused pre-TCRα^+^ T cells in a NOG mouse xenograft model [93].

More strategies have been developed to avoid disrupting the TCR. An example is the study by Michaux et al. who introduced a gene encoding a truncated CD3ζ peptide that competes with endogenous CD3ζ for the formation of the TCR complex, giving attenuated TCR responses and avoiding GvHD [49].

Another approach is to reduce the TCR complex formation by the expression of a protein expression blocker which retains CD3ε [50]. Although TCR elimination would be avoided, in this case the strategy would not allow CD3-mediated T cell activation.

### Selection of specific cell sources/subpopulations for allogeneic CAR-T cell production

The use of T cells obtained from a compatible donor as starting material for allogeneic CAR-T cell manufacturing is a technically simple strategy. However, it depends on donor availability and maintains the main disadvantages of autologous therapies, such as high cost, lack of standardisation and manufacturing time. It is not a truly “off-the-shelf” therapy, but has been used experimentally in some patients [55, 97]. Additionally, several studies investigate the use of specific cell sources or subpopulations to produce “off-the-shelf” CAR-T cell therapies. In the following, we describe these different strategies that in most cases are based on the selection of a specific subpopulation that would allow allogeneic use without causing GvHD.

#### γδ T cells

While most human T cells are αβ T cells, γδ T cells represent only 1–5% of circulating T cells, although they are prevalent in some epithelial tissues [65]. These cells are involved in the innate immunity, but are also modulators in the adaptive immunity and can target tumour cells indirectly [63]. They are involved in antitumour immunity and tumour surveillance and their tumour infiltration is highly correlated with patient survival [62]. Interestingly, it has been observed in vitro that γδ T cells are able to target tumour-associated macrophages, which are one of the main agents of tumour microenvironment-immunosuppression [69]. γδ T cells express TCRγδ and recognise their target cells in an HLA-independent manner leading to low or no risk of GvHD, so they can be used for allogeneic clinical application without eliminating TCR expression or signalling [62, 64]. Specifically, the TCRγδ recognises native unprocessed antigens, while TCRαβ (characteristic of αβ T cells) recognises processed peptide antigens presented by HLA molecules [62]. In addition to the targets recognised by TCRγδ, these cells can recognise stressed, damaged, infected and malignant cells through NK-specific receptors, in particular the natural killer group 2D receptor (NKG2D) [62, 67].

γδ T cells can be classified into two main subsets based on their Vδ chains: Vδ1 and Vδ2. Vδ2 T cells are enriched in circulating peripheral blood, while Vδ1 T cells are predominantly tissue-resident [63], although they also represent 12–22% of total circulating γδ T cells [62]. Zhai et al. used for CAR-T cell production the Vγ9Vδ2 T cells, a subtype of Vδ2, which is the main subset in peripheral blood and, in addition, they can act as antigen-presenting cells (APC) after their activation, which may play a key role in enhancing the immune response. In this study, the authors demonstrate that CAR-Vγ9Vδ2 T cells have similar or stronger cytotoxic effects than common CAR-T cells (based on αβ T cells), although their cytotoxic capacity in vitro was less persistent [65]. On the other hand, Vδ1 T cells are also used as sources for allogeneic CAR-T cells because of their naïve-like memory phenotype, tissue tropism and reduced susceptibility to activation-induced cell death [68]. This subset demonstrated tumour growth inhibition and absence of GvHD in immunodeficient mice [62]. Furthermore, after expansion, they presented a mainly naïve-like memory phenotype with low exhaustion level [67].

The main problem with these cells, apart from their low levels in human tissues, is the difficulty of in vitro expansion, so different strategies are being studied in this regard. Interestingly, it has been found that the donors’ healthy lifestyle and physical exercise immediately prior to PBMCs extraction correlate with higher *ex vivo* expansion of γδ T cells. The same authors reported also that interleukin (IL)-21 increased proliferation of γδ T cells that did not show proliferation in culture at first [63]. Other studies expose the cells to zoledronate for both in vitro and in vivo expansion of γδ T cells [64]. To expand a specific subset like Vδ1, Polito et al. co-cultured the γδ T cells with artificial APCs, irradiated and modified to express CD86/41BBL/CD40L and the cytomegalovirus (CMV)-antigen-pp65. These APCs were designed to express also an inducible suicide gene that allows to eliminate them from the final product [68]. Ferry et al. also expanded Vδ1 T cells by stimulating TCRαβ- and CD56-depleted PBMCs with anti-CD3 and IL-15 [70]. Another strategy to expand this subpopulation is based on an agonistic monoclonal antibody directed against the Vδ1 chain [67]. An anti-CD20 Vδ1 γδ CAR-T cell product manufactured using this method has shown very good results in B cell lymphoma animal models and is being tested in phase I clinical trials [62].

A different approach assayed with γδ T cells is based on the transduction of a non-signalling CAR (NSCAR), which lacks the intracellular activator domain, thus serving only to direct the therapeutic cells to the target cells, but the antitumour effect is performed through their inherent cytotoxic activity. These cells showed an increased cytotoxic activity compared to naïve cells [66].

#### Induced pluripotent stem cells (iPSCs)

iPSCs have an unlimited proliferation capacity, while maintaining their pluripotency and lineage differentiation potential [72]. Therefore, a bank of iPSC lines with different homozygous HLA combinations could be generated that could reduce the risk of immune rejection of CAR-T cells derived from them, by choosing the optimal iPSC source according to HLA matching between host and graft. Another option is the use of gene editing to eliminate HLA-I and/or HLA-II expression, as described in previous sections, which must be combined with TCR elimination to avoid GvHD [73, 75, 77]. An advantage of using iPSCs is that CAR-T cells can be generated from a single iPSC clone with the capacity for clonal expansion and therefore the genetic modifications they undergo would be homogeneous in the final cell population [72]. However, the quality controls should be strict because undifferentiated proliferating iPSCs may compromise product safety, since they could induce important adverse effects such as teratomas [98].

iPSCs can be developed from different cell types, such as fibroblasts or lymphocytes, that are reprogrammed into a less differentiated cell by inducing the expression of specific factors. For example, Wang et al. generated iPSCs from naïve or memory T cells (CD62L^+^) using the following factors: KLF4, OCT3/4, SOX2, LIN28, L-MYC, and shRNA for TP53. Selected iPSCs clones are transduced with the CAR, and CAR-expressing cells are expanded and subsequently differentiated into T cells, progressing consecutively into mesoderm cells, haematopoietic cells and finally T cells [72]. Other studies even reprogrammed γδ T cells into iPSCs as an attempt to avoid the risk of GvHD [71] or generated iPSCs from T cells with precise TCR specificity to recognise tumour cells through both the CAR and the TCR [74]. However, van der Stegen et al. observed that constitutive CAR expression in T cell-derived iPSCs could interfere in the T cell maturation by acquiring an innate phenotype, so they delayed and regulated the CAR expression and modulated its signalling strength. In this way, they induced CAR-mediated T cell maturation obtaining TCR^−^ CD8αβ^+^ CAR-T cells, which are similar to CD8αβ^+^ CAR-T cells from PBMCs, and achieved tumour control in vivo without GvHD [77].

To solve the problem of immune rejection, Wang et al. produced iPSC-derived CAR-T cells that lack not only HLA-I and HLA-II, but also the poliovirus receptor CD155, which encodes a ligand for the NK cell-activating receptor DNAM-1. Moreover, they also induced the expression of HLA-E, thus preventing rejection by NKG2A^+^ NK cells, as these cells eliminate HLA-I^−^ T cells [75].

#### Umbilical cord blood T cells

Another source of T cells for allogeneic use is umbilical cord blood (UCB), which is an enriched source of haematopoietic stem cells (HSCs). Access to this source would be easy as there are large numbers of umbilical cord samples cryopreserved in cell banks; however, there is a limited total number of HSCs in each UCB [78, 79]. HSCs are able to self-renew and expand *ex vivo*, and to differentiate into T cells, at higher orders than in the manufacture of autologous T cells. Furthermore, Boyd et al. obtained from UCB a high percentage of γδ T cells, which may be caused by the lack of thymic cortical epithelial cells, which are necessary for positive TCRαβ selection. Producing large numbers of these cells has many advantages as mentioned above and, in addition, selection could eliminate TCRαβ^+^ T cells, which are the major culprits of both GvHD and autoimmunity [78]. In other study, Liu et al. saw that CAR-T cells from UCB had higher naïve T cell proportions and longer tumour suppression in vivo than CAR-T cells derived from patient peripheral blood. In addition, this product released lower levels of IL-10 and contained a lower proportion of Tregs, which is indicative of better efficacy [80].

Van Caeneghem et al. selected CD34^+^ HSCs from cord blood and expanded them with OP9-DL1 feeder cells. After being T-lineage committed, they transduced the CAR gene and continued the culture with OP9-DL1 cells, differentiating them into T cells. These CAR-T cells had a naïve cell phenotype (CD45RA^+^ CD62L^+^) and also lacked membrane-expressed TCRαβ complexes, thus reducing the risk of GvHD. The authors explained that this is because CAR expression suppressed TCRβ rearrangements and drastically reduced TCRα rearrangements. Therefore, these CAR-T cells effectively eliminate tumour cells, while reducing the induction of GvHD [79].

#### Memory T cell subpopulations

An easier approach to achieve allogeneic CAR-T cell therapy is the use of T cells with a specific memory phenotype. It is considered that their TCR has a specificity directed to previously detected antigens, which are expected to be different from those of the patient receiving the CAR-T cell therapy. This non-alloreactive TCR together with the fact that they traffic less to organs that manifest GvHD, such as the gastrointestinal tract, leads to memory T cells being less prone to develop GvHD [83].

CAR-T cell manufacturing processes, including those for autologous products, have been optimized to increase enrichment in early memory T cell populations (i.e. Tscm and central memory T cells, Tcm) because they possess better proliferative and stemness potential than more differentiated T cells [99] and have demonstrated high expansion and persistence in vivo after adoptive T cell transfer [100, 101]. A common strategy to achieve this enrichment is the use of IL-7 and IL-15 instead of IL-2 for culturing supplementation, which has also demonstrated to confer a higher antitumour response to CAR-T cells [102]. Additionally, since T cell differentiation subsets possess different metabolic demands, it has been proposed to reprogram CAR-T cell metabolism in order to modify the proportion of memory T cell subsets in the final product, thus aiming to influence its antitumour activity [103].

In contrast to the aforementioned characteristics of memory T cells, Tn have high alloreactivity potential and effector T cells have low persistence in vivo, both being CD45RA^+^ T cells [82, 83]. Therefore, to develop allogeneic CAR-T cells based on memory T cell selection, most efforts to date have focused on depleting CD45RA^+^ T cells, since this strategy allows to eliminate both Tn and differentiated effector T cells and to obtain a purified population of Tcm and effector memory T cells (Tem). Fernández et al. followed this purification strategy using magnetic bead-based technology under GMP conditions, and they proved that the infusion of this CAR-T cell product was safe in animal models [83]. Moreover, Kim-Hoehamer et al. included an additional CD14^+^ depletion step to eliminate monocytes starting from PBMCs. This study also showed that selected memory T cells are able to respond to virus after *ex vivo* expansion, thus maintaining their antiviral activity, which is also beneficial to the patient, especially to treat virus-induced tumours. Due to its good in vivo results, this CAR-T CD45RA^−^/CD14^−^ cell therapy has received FDA approval for clinical trials [81].

Some authors included selection with additional markers directed to specifically isolate the Tcm subpopulation (CD45RA^−^/CD62L^+^), since it shows increased persistence in vivo than Tem. For example, Wang et al. selected CD8^+^ Tcm cells to manufacture a new CAR-T cell product that is being currently investigated in a phase I/II clinical trial [82]. However, even when selecting the Tcm subpopulation initially, there are significant numbers of Tem after culture [82], but this occurs to a greater extent when they are not selected [81, 83]. Regarding the CD4:CD8 ratio, although some studies initially focused on purifying the cytotoxic CD8^+^ T cells, such as the aforementioned by Wang et al. [82], many evidences revealed the important role of CD4^+^ T cells in the antitumour effect of CAR-T cell products, since even isolated CD4^+^ CAR-T cells have demonstrated to exert a strong antitumour activity through a mechanism mainly based on interferon (IFN)-γ production [104].

#### Virus-specific T cells

Virus-specific T (VST) cells have a TCR that specifically recognises viral antigens such as CMV, Epstein-Barr virus (EBV), Varicella Zoster virus (VZV), Adenovirus (AdV) or Influenza [84, 85, 87–91]. Infusion of VST cells has already been used in patients with Hodgkin lymphoma -almost 40% of whom express EBV-associated antigens in their tumour cells- with good tolerance and remission rates. This infection increases the proliferation of the VST cells through stimulation of their TCR, thus promoting the elimination of the EBV-infected memory B cells that remain lifelong in lymphoid tissues. Therefore, the addition of a specific CAR avoids resistance to EBV-specific T cells as well as tumour relapse by immune escape due to EBV antigen loss, thus improving the antitumour effect [85]. A CAR-VST cell product with HER2-specificity has been already proved in autologous use in a phase I clinical trial, being safe and clinically beneficial for patients with progressive glioblastoma with CMV seropositivity [86]. Importantly, given the specificity of their TCR towards different viral antigens, these therapeutic cells are potentially not alloreactive and could be used as a cell source for allogeneic CAR-T cell products. Moreover, the specific viral antigens can be used to enhance their activation, proliferation and persistence [86, 91].

To obtain VST cells in vitro, PBMCs are exposed to dendritic cells pulsed with different virus-specific peptides [84, 90] or autologous virus-transformed B lymphoblastoid cell lines, as some studies did for EBV [85, 88, 91]. They can be also co-cultured with autologous T cells that are pulsed with a viral antigen vector or viral peptide pools to express one [89] or more viral antigens [87], respectively. Thus, these T cells can target different antigens of one [85, 88, 89, 91], two [84, 90] or more types of viruses [87].

As previously mentioned, some of the problems with allogeneic CAR-T cells include that they have low persistence and do not proliferate sufficiently after infusion, especially in solid tumours, which have an immunosuppressive microenvironment. With the CAR-VST cells, one of the ways to increase this proliferation and function in vivo is through their viral-specific TCR. Thus, in the absence of viral re-activation or new infection, viral vaccines or oncolytic viruses can be used to boost CAR-VST cells, since they polarise immunity towards Th1 and enhance the activity of the therapeutic cells [84]. It has been reported, for example, the enhancement of in vivo antitumour activity of CMV-CD19 bi-specific CAR T cells -manufactured by transducing CMV-specific T cells with an anti-CD19 CAR- by the administration of anti-CMV vaccination. This strategy would also allow to reduce *ex vivo* proliferation, which contributes to T cell exhaustion, to increase CAR-T cell persistence and duration of response, and to induce re-stimulation of T cells in vivo in case of tumour relapse [105]. Moreover, the infusion of irradiated APCs expressing the viral antigen recognised by the endogenous TCR of the CAR-VST cells also induce an increase in activation, proliferation, and cytokine secretion and, consequently, enhance the antitumour activity of the therapeutic cells [89]. Another strategy to augment CAR-VSTs expansion in vivo is to transfect an IL-7 receptor, which is not expressed on Treg cells, so that IL-7 administration would activate CAR-T cells and increase their proliferation and antitumour activity even in the presence of Treg cells [91]. In line with increasing in vivo persistence, Omer et al. have used VSTs with a co-stimulatory CAR (CoCAR), which lacks the CD3ζ domain. Thus, the CoCAR is responsible for inducing co-stimulatory signals, when it detects the target antigen, and a survivin-specific transgenic TCR is responsible for the first signal of T cell activation. In this way, they modulate cytotoxicity, reduce potential on-target/off-tumour toxicity of the CAR and enhance persistence and antitumour activity of the CoCAR-VST cells [90].

#### Cytokine-induced killer cells

Cytokine-induced killer (CIK) cells are effector T cells with acquired NK-like cytotoxicity. They express T cell markers and contain a high proportion of NK-like T cells (CD3^+^CD56^+^). Generation of these cells is performed by *ex vivo* culture of PBMCs, usually in presence of IL-2, IFN-γ, and anti-CD3 monoclonal antibody. CIK cells have the advantage of exhibiting non-HLA-restricted cytotoxicity and very low alloreactivity, but the heterogeneity of the cellular product obtained with this procedure is an important disadvantage. Magnani et al. produced CAR-CIK cells using the Sleeping Beauty transposon plasmid system to introduce the CAR and these cells were exposed to irradiated PBMCs from the same source to avoid cell death caused by the non-viral transfection, allowing effective gene transfer and efficient T cell expansion. This CAR-CIK final product consists of a heterogeneous T cell population; however, it induced a highly competent T cell response with antitumour activity in vivo, and the maintenance of memory phenotype might indicate that this response could be early and sustained [92]. Nevertheless, the clinical trial showed that the CAR-CIK product had moderate persistence, since it ranged from 22 to 300 days after therapy administration, with a median duration of 94 days [106]. This could be explained, at least in part, by the low proportion of early memory T cells in the final product [92]. Indeed, some authors demonstrated that CIK phenotype is very similar to that described for the terminally differentiated memory T cell subset [107], which possess less proliferation and persistence capacity in vivo.

## Discussion

In this systematic review we have attempted to compile, by means of a systematic search and following the PRISMA guideline standards [33], all the reported strategies for the development of CAR-T cells from allogeneic sources, designed so that they do not produce GvHD and are not rejected by the recipient.

CAR-T cell immunotherapy has arisen as a novel therapeutic strategy that has shown surprising efficacy in the treatment of B cell neoplasms [20]. It has a very high response rate over a prolonged period of time, leading to tumour control and remission for several years in some cases [26]. However, there are certain adverse effects of CAR-T therapy to be aware of, such as CRS and ICANS [22]. CAR-T cell efficacy lies not only in the structure of the CAR, by which it can recognise specific surface antigens, designed to direct the cytotoxic action on target cells and to minimise the risk of toxicity to healthy tissues; but also, in the nature/characteristics of the specific T cell source used as starting material and the different metabolism, expansion capacity, persistence and memory phenotype of the product CAR-T cells [2].

So far, all CAR-T cell therapies approved for commercialisation, as well as those tested in most clinical trials, are autologous CAR-T cells [4, 6–10]. Autologous treatments use the patient’s own cells and are therefore, a priori, safer and more effective than allogeneic therapies, as they are not immunogenic and persist in vivo for a longer term [26]. However, they depend on the quality of the patient’s own T cells, require a manufacturing time from apheresis to infusion that can be critical for the patient, their standardization is limited by differences in patient starting material, and large-scale manufacturing would only be useful for retreating the same patient [25, 27]. In contrast, allogeneic CAR-T cell treatments are potentially alloreactive and immunogenic, as they have the risk to cause GvHD and their efficacy is conditioned by host immune rejection [26, 31]. However, they have the advantages of possible standardisation and large-scale manufacturing, production from optimal cell sources, theoretically lower manufacturing costs, and allowing immediate treatment of the patient and even re-dosage if necessary [26, 27]. In addition, an important advantage of allogeneic over autologous products is that contamination of the product with the patient’s tumour cells is avoided, since a case has been described in which a neoplastic cell was transduced with an anti-CD19 CAR, masking the cell’s own CD19 molecules and becoming resistant to therapy [108].

As we have seen, there are different approaches to developing allogeneic CAR-T cell therapies. One of the methods is the genetic modification (additional to CAR transgene introduction) of T cells obtained from healthy donors to remove TCR and HLA-I/-II molecules [48]. Among the different methodologies, CRISPR/Cas9 technology is the most commonly used approach due to low rate of off-target mutagenesis, simplicity and accessibility [48]. However, TCR elimination may pose a problem for *ex vivo* cell expansion, as this strategy prevents the expression of the membrane CD3 subunit, involved in T cell stimulation signalling and by which the cells are usually activated *ex vivo* to induce their proliferation [93]. Another problem is that CAR-T HLA^−^ cells can be recognised and eliminated by NK cells [75]. One assayed strategy to avoid this is the introduction of the B2M-HLA-E and B2M-HLA-G fusion proteins, which bind to inhibitory NK cell receptors NKG2A and KIR2DL4 as well as LILRB1, respectively. The authors described that the constitutive expression of these fusion proteins in CAR-T cells prevented allogeneic NK cell-mediated lysis [95]. It is important to consider that the genetically modified products have to go through a process of high regulation and quality controls to check for off-target effects and genetic rearrangements because gene editing tools may act on untargeted genomic sites and create cleavages that potentially produce adverse safety consequences [109].

The alternative to additional genetic modifications to produce allogeneic CAR-T cells is to use a low alloreactive T cell source or subpopulation. One of them are γδ T cells, which have a cytotoxic phenotype and their ability to infiltrate tissues makes them suitable for the treatment of both haematological and solid tumours [62, 63]. In the latter, common CAR-T cell therapy based on αβ T cells has been less effective, so their development would be of great interest [67]. Moreover, they are easy to manufacture, recognise antigens independently of HLA and have NK cell receptors, although their availability is low and their *ex vivo* proliferation capacity may be limited [62, 64, 67]. CIK cells also have a cytotoxic phenotype similar to NK cells, non-HLA-restricted cytotoxicity and therefore little alloreactivity, making them another interesting option to be used in allogeneic CAR-T cell therapy; however, it is a less explored strategy, probably due to the high cell heterogeneity in the final product, with only one reference identified in this systematic review [92]. VST cells have a straightforward production and a non-alloreactive TCR that specifically recognises viral antigens, thus also conferring protection against viral infections, which may be advantageous in immunocompromised patients and in the treatment of virus-induced cancers [91]. They have been effective and safe in neoplasms such as Hodgkin’s lymphoma associated with EBV infection, but their expansion and persistence in vivo is low [85]. In order to increase them, some studies use commercially available virus vaccines, among other strategies, to boost the virus-specific T cells after infusion of the CAR-T cell product, improving also the tumour control [110, 111]. Regarding memory T cells, Tcm and Tem (both CD45RA^−^) are easy to obtain and have low alloreactive TCRs, usually with antiviral activity, which makes them an interesting option to develop allogeneic CAR-T cell products [81, 83]. Additionally, Tscm cells are gaining relevance because these cells are less differentiated and have more self-renewal capacity than Tcm and Tem cells [82]. In vivo CAR-T cell studies have demonstrated that this subpopulation show higher proliferation, longer persistence, stronger antitumour activity, and lower exhaustion profile, and that it is less prone to induce CRS, in comparison with unselected CAR-T cells [112].

Allogeneic T cells can also be obtained from UBC, since some authors demonstrated that the HSCs-derived T cells lacked surface-expressed TCRαβ complexes, so they have high immune tolerance and low incidence of GvHD; however, UBC has a limited number of T cells [78, 79]. Furthermore, another source is the iPSCs which are probably the most complex to produce, as they also require additional modifications, and the product should be carefully analysed in order to avoid undifferentiated iPSCs, unwanted genetic modifications and consequent safety issues [73, 75, 98]. However, they have great potential given their infinite capacity for self-renewal and clonal expansion, which would result in a fully homogeneous product [72]. Once gene editing is optimised, it could become a truly universal treatment.

Finally, an important issue in the production of allogeneic CAR-T cells, also relevant for autologous CAR-T cell products, is the establishment of manufacturing processes that achieve a high standardisation of the final products, ideally making them more efficient and less operator-dependent, as with the use of specific platforms developed for this purpose [57, 83].

## Conclusions

In conclusion, allogeneic CAR-T cell therapy has great advantages over autologous CAR-T cell therapy due to the possibilities of large-scale manufacturing and the consequent potential cost reduction, greater standardisation of the product, better quality of the therapeutic cells, as well as immediate availability of the product to treat the patients. In recent years, a wide range of different approaches have been studied to achieve the production of allogeneic CAR-T cell therapies, which could be classified into two main categories: those involving extra genetic modifications, in addition to CAR transgene introduction, and those relying on the selection of alternative cell sources/subpopulations as starting material. The latter group encompasses novel strategies, many of which have been reported in the last 5 years, including the use as cell sources of γδ T cells, iPSCs, UCB T cells, memory T cells subpopulations, VST cells and CIK cells. Although genetic modification of T cells is the most widely used approach, new strategies combining both methods have emerged. However, further preclinical and clinical research is needed to stablish the most appropriate strategy for the production of allogeneic CAR-T cells, which should minimise the major risks of this therapy: GvHD and immune rejection. The commercialisation of this kind of promising antitumour therapy could extend the availability of CAR-T cells to a larger number of patients.

## Data Availability

Not Applicable.

## Abbreviations

AAV: Adeno-associated virus
AdV: Adenovirus
AEMPS: Spanish Agency of Medicines and Medical Devices
ALL: Acute lymphoblastic leukemia
APC: Antigen-presenting cells
ATMPs: Advanced therapy medicinal products
B2M: β2-microglobulin
BCMA: B cell maturation antigen
CAR: Chimeric antigen receptor
CIK: Cytokine-induced killer
CMV: Cytomegalovirus
CoCAR: Co-stimulatory CAR
CRS: Cytokine release syndrome
CTLA-4: Cytotoxic T-lymphocyte antigen 4
dCK: Deoxycytidine kinase
DLBCL: Diffuse large B cell lymphoma
EBV: Epstein-Barr virus
EGFRt: Truncated epidermal growth factor receptor
EMA: European Medicines Agency
FDA: Food and Drug Administration
GvHD: Graft-versus-host disease
HLA: Human leukocyte antigen
HLA-I: HLA class I
HLA-II: HLA class II
HSC: Haematopoietic stem cells
ICANS: Immune effector cell-associated neurotoxicity syndrome
IFN: Interferon
IL: Interleukin
iPSCs: Induced pluripotent stem cells
LBCL: Large B cell lymphoma
MCL: Mantle cell lymphoma
NK: Natural killer
NKG2D: Natural killer group 2D receptor
NSCAR: Non-signalling CAR
PBMC: Peripheral blood mononuclear cells
PD1: Programmed cell death 1
PRISMA: Preferred Reporting Items for Systematic Reviews and Meta-Analyses
r/r: Relapsed or refractory
scFv: Antibody-derived single-chain variable fragment
TALEN: Transcription activator-like effector nucleases
Tcm: Central memory T cells
TCR: T cell receptor
Tem: Effector memory T cells
Tregs: Regulatory T cells
Tscm: Stem cell memory T cells
UCB: Umbilical cord blood
VST: Virus-specific T
VZV: Varicella Zoster Virus
ZFNs: Zinc finger nucleases

## References

[1] Sterner RC, Sterner RM. CAR-T cell therapy: current limitations and potential strategies. Blood Cancer J. 2021;11–69. 10.1038/s41408-021-00459-7 PMC8024391 33824268

[2] Larson RC, Maus MV (2021). Recent advances and discoveries on the mechanisms and functions of CAR T cells. Nat Rev Cancer.

[3] Huang R, Li X, He Y, Zhu W, Gao L, Liu Y (2020). Recent advances in CAR-T cell engineering. J Hematol Oncol.

[4] KYMRIAH (tisagenlecleucel) | FDA. 2022. https://www.fda.gov/vaccines-blood-biologics/cellular-gene-therapy-products/kymriah-tisagenlecleucel. Accessed 13 Oct 2022.

[5] Kymriah | European Medicines Agency. 2022. https://www.ema.europa.eu/en/medicines/human/EPAR/kymriah. Accessed 21 Dec 2022.

[6] YESCARTA (axicabtagene ciloleucel) | FDA. 2022. https://www.fda.gov/vaccines-blood-biologics/cellular-gene-therapy-products/yescarta-axicabtagene-ciloleucel. Accessed 13 Oct 2022.

[7] TECARTUS (brexucabtagene autoleucel) | FDA. 2022. https://www.fda.gov/vaccines-blood-biologics/cellular-gene-therapy-products/tecartus-brexucabtagene-autoleucel. Accessed 13 Oct 2022.

[8] BREYANZI (lisocabtagene maraleucel) | FDA. 2022. https://www.fda.gov/vaccines-blood-biologics/cellular-gene-therapy-products/breyanzi-lisocabtagene-maraleucel. Accessed 13 Oct 2022.

[9] ABECMA (idecabtagene vicleucel) | FDA. 2022. https://www.fda.gov/vaccines-blood-biologics/abecma-idecabtagene-vicleucel. Accessed 13 Oct 2022.

[10] CARVYKTI | FDA. 2022. https://www.fda.gov/vaccines-blood-biologics/carvykti. Accessed 14 Oct 2022.

[11] Trias E, Juan M, Urbano-Ispizua A, Calvo G (2022). The hospital exemption pathway for the approval of advanced therapy medicinal products: an underused opportunity? The case of the CAR-T ARI-0001. Bone Marrow Transplant.

[12] Ogasawara K, Lymp J, Mack T, Dell’Aringa J, pin Huang C, Smith J (2022). In vivo Cellular Expansion of Lisocabtagene Maraleucel and Association with Efficacy and Safety in Relapsed/Refractory large B-Cell lymphoma. Clin Pharmacol The.

[13] Boardman DA, Philippeos C, Fruhwirth GO, Ibrahim MAA, Hannen RF, Cooper D (2017). Expression of a chimeric Antigen receptor specific for Donor HLA Class I enhances the potency of Human Regulatory T cells in preventing human skin transplant rejection. Am J Transplant.

[14] Muller YD, Ferreira LMR, Ronin E, Ho P, Nguyen V, Faleo G (2021). Precision Engineering of an Anti-HLA-A2 chimeric Antigen receptor in Regulatory T cells for Transplant Immune Tolerance. Front Immunol.

[15] Tenspolde M, Zimmermann K, Weber LC, Hapke M, Lieber M, Dywicki J (2019). Regulatory T cells engineered with a novel insulin-specific chimeric antigen receptor as a candidate immunotherapy for type 1 diabetes. J Autoimmun.

[16] Martin A, Daris M, Johnston JA, Cui J (2021). HLA-A*02:01-directed chimeric antigen receptor/forkhead box P3-engineered CD4 + T cells adopt a regulatory phenotype and suppress established graft-versus-host disease. Cytotherapy.

[17] Rafei H, Kantarjian HM, Jabbour EJ (2019). Recent advances in the treatment of acute lymphoblastic leukemia. Leuk Lymphoma.

[18] Hunger SP, Raetz EA (2020). How I treat relapsed acute lymphoblastic leukemia in the pediatric population. Blood.

[19] He J, Chen Z, Xue Q, Sun P, Wang Y, Zhu C (2022). Identification of molecular subtypes and a novel prognostic model of diffuse large B-cell lymphoma based on a metabolism-associated gene signature. J Transl Med.

[20] Meng J, Wu XQ, Sun Z, Xun R, De, Liu MS, Hu R (2021). Efficacy and safety of CAR-T cell products Axicabtagene Ciloleucel, Tisagenlecleucel, and Lisocabtagene Maraleucel for the treatment of hematologic malignancies: a systematic review and Meta-analysis. Front Oncol.

[21] Bachy E, Le Gouill S, Di Blasi R, Sesques P, Manson G, Cartron G (2022). A real-world comparison of tisagenlecleucel and axicabtagene ciloleucel CAR T cells in relapsed or refractory diffuse large B cell lymphoma. Nat Med.

[22] Alexander M, Culos K, Roddy J, Shaw JR, Bachmeier C, Shigle TL (2021). Chimeric Antigen receptor T cell therapy: a comprehensive review of clinical efficacy, toxicity, and best Practices for Outpatient Administration. Transpl Cell Ther.

[23] Flugel CL, Majzner RG, Krenciute G, Dotti G, Riddell SR, Wagner DL (2023). Overcoming on-target, off-tumour toxicity of CAR T cell therapy for solid tumours. Nat Rev Clin Oncol.

[24] Choi BD, Yu X, Castano AP, Darr H, Henderson DB, Bouffard AA (2019). CRISPR-Cas9 disruption of PD-1 enhances activity of universal EGFRvIII CAR T cells in a preclinical model of human glioblastoma. J Immunother Cancer.

[25] Sommer C, Boldajipour B, Kuo TC, Bentley T, Sutton J, Chen A (2019). Preclinical evaluation of allogeneic CAR T cells targeting BCMA for the treatment of multiple myeloma. Mol Ther.

[26] Depil S, Duchateau P, Grupp SA, Mufti G, Poirot L (2020). Off-the-shelf’ allogeneic CAR T cells: development and challenges. Nat Rev Drug Discovery.

[27] Harrison RP, Zylberberg E, Ellison S, Levine BL (2019). Chimeric antigen receptor–T cell therapy manufacturing: modelling the effect of offshore production on aggregate cost of goods. Cytotherapy.

[28] Lacey SF, Xu J, Ruella M, Barrett DM, Kulikovskaya I, Ambrose DE (2016). Cars in leukemia: relapse with Antigen-Negative leukemia originating from a single B cell expressing the leukemia-targeting CAR. Blood.

[29] Engels B, Zhu X, Yang J, Price A, Sohoni A, Stein AM (2021). Preservation of T-Cell stemness with a Novel Expansionless CAR-T Manufacturing process, which reduces Manufacturing Time to Less Than two days, drives enhanced CAR-T cell efficacy. Blood.

[30] Flinn IW, Jaeger U, Shah NN, Blaise D, Briones J, Shune L (2021). A first-in-human study of YTB323, a Novel, Autologous CD19-Directed CAR-T cell therapy manufactured using the novel T-Charge TM platform, for the treatment of patients (pts) with Relapsed/Refractory (r/r) diffuse large B-Cell lymphoma (DLBCL). Blood.

[31] Martínez Bedoya D, Dutoit V, Migliorini D, Allogeneic CART, Cells (2021). An alternative to Overcome Challenges of CAR T Cell Therapy in Glioblastoma. Front Immunol.

[32] Morgan MA, Büning H, Sauer M, Schambach A (2020). Use of Cell and Genome Modification Technologies to generate improved “Off-the-Shelf” CAR T and CAR NK cells. Front Immunol.

[33] Page MJ, Mckenzie JE, Bossuyt PM, Boutron I, Hoffmann TC, Mulrow CD (2021). The PRISMA 2020 statement: an updated guideline for reporting systematic reviews. BMJ.

[34] Valton J, Guyot V, Marechal A, Filhol JM, Juillerat A, Duclert A (2015). A multidrug-resistant Engineered CAR T cell for allogeneic combination immunotherapy. Mol Ther.

[35] Ren J, Zhang X, Liu X, Fang C, Jiang S, June CH (2017). A versatile system for rapid multiplex genome-edited CAR T cell generation. Oncotarget.

[36] Lee J, Sheen JH, Lim O, Lee Y, Ryu J, Shin D (2020). Abrogation of HLA surface expression using CRISPR/Cas9 genome editing: a step toward universal T cell therapy. Sci Rep.

[37] Hu Y, Zhou Y, Zhang M, Ge W, Li Y, Yang L (2021). CRISPR/Cas9-engineered universal CD19/CD22 dual-targeted CAR-T cell therapy for relapsed/refractory B-cell acute lymphoblastic leukemia. Clin Cancer Res.

[38] Gautron AS, Juillerat A, Guyot V, Filhol JM, Dessez E, Duclert A (2017). Fine and predictable tuning of TALEN Gene Editing Targeting for Improved T Cell Adoptive Immunotherapy. Mol Ther Nucleic Acids.

[39] Kagoya Y, Guo T, Yeung B, Saso K, Anczurowski M, Wang CH (2020). Genetic ablation of HLA class I, class II, and the T-cell receptor enables allogeneic T cells to be used for adoptive T-cell therapy. Cancer Immunol Res.

[40] Georgiadis C, Preece R, Nickolay L, Etuk A, Petrova A, Ladon D (2018). Long terminal repeat CRISPR-CAR-Coupled “Universal” T cells mediate Potent Anti-leukemic Effects. Mol Ther.

[41] Poirot L, Philip B, Schiffer-Mannioui C, Le Clerre D, Chion-Sotinel I, Derniame S (2015). Multiplex genome-edited T-cell manufacturing platform for “off-the-shelf” adoptive T-cell immunotherapies. Cancer Res.

[42] Siler Panowski AH, Srinivasan S, Tan N, Tacheva-Grigorova SK, Smith B, Mak YS (2022). Preclinical development and evaluation of allogeneic CAR T cells targeting CD70 for the treatment of renal cell carcinoma. Cancer Res.

[43] Sommer C, Cheng HY, Nguyen D, Dettling D, Yeung YA, Sutton J (2020). Allogeneic FLT3 CAR T cells with an off-switch exhibit potent activity against AML and can be depleted to Expedite Bone Marrow Recovery. Mol Ther.

[44] Juillerat A, Tkach D, Yang M, Boyne A, Valton J, Poirot L (2020). Straightforward Generation of Ultrapure off-the-Shelf allogeneic CAR-T cells. Front Bioeng Biotechnol.

[45] Stenger D, Stief TA, Kaeuferle T, Willier S, Rataj F, Schober K (2020). Endogenous T-cell receptor promotes in vivo persistence of CD19-CAR-T cells compared to a CRISPR/Cas9-mediated T-cell receptor knockout CAR. Blood.

[46] Li S, Wang X, Yuan Z, Liu L, Luo L, Li Y (2021). Eradication of T-ALL cells by CD7-targeted universal CAR-T cells and initial test of ruxolitinib-based CRS management. Clin Cancer Res.

[47] Cooper ML, Choi J, Staser K, Ritchey JK, Devenport JM, Eckardt K (2018). An ‘off-the-shelf’ fratricide-resistant CAR-T for the treatment of T cell hematologic malignancies. Leukemia.

[48] Ren J, Liu X, Fang C, Jiang S, June CH, Zhao Y (2017). Multiplex genome editing to generate universal CAR T cells resistant to PD1 inhibition. Clin Cancer Res.

[49] Michaux A, Mauën S, Breman E, Dheur M-S, Twyffels L, Saerens L (2022). Clinical Grade manufacture of CYAD-101, a NKG2D-based, First in Class, non–gene-edited allogeneic CAR T-Cell therapy. J Immunother.

[50] Kamiya T, Wong D, Png YT, Campana D (2018). A novel method to generate T-cell receptor–deficient chimeric antigen receptor T cells. Blood Adv.

[51] Wang X, Cabrera FG, Sharp KL, Spencer DM, Foster AE, Bayle JH (2021). Engineering Tolerance toward Allogeneic CAR-T cells by regulation of MHC surface expression with human herpes Virus-8 proteins. Mol Ther.

[52] Hale M, Lee B, Honaker Y, Leung WH, Grier AE, Jacobs HM (2017). Homology-Directed recombination for enhanced Engineering of chimeric Antigen receptor T cells. Mol Ther Methods Clin Dev.

[53] MacLeod DT, Antony J, Martin AJ, Moser RJ, Hekele A, Wetzel KJ (2017). Integration of a CD19 CAR into the TCR Alpha Chain Locus streamlines production of allogeneic gene-edited CAR T cells. Mol Ther.

[54] Yang M, Tkach D, Boyne A, Kazancioglu S, Duclert A, Poirot L (2022). Optimized two-step electroporation process to achieve efficient nonviral‐mediated gene insertion into primary T cells. FEBS Open Bio.

[55] Zhang JP, Zhang R, Tsao ST, Liu YC, Chen X, Lu DP (2018). Sequential allogeneic and autologous CAR-T–cell therapy to treat an immune-compromised leukemic patient. Blood Adv.

[56] Webber BR, Lonetree C, lin, Kluesner MG, Johnson MJ, Pomeroy EJ, Diers MD (2019). Highly efficient multiplex human T cell engineering without double-strand breaks using Cas9 base editors. Nat Commun.

[57] Alzubi J, Lock D, Rhiel M, Schmitz S, Wild S, Mussolino C (2021). Automated generation of gene-edited CAR T cells at clinical scale. Mol Ther Methods Clin Dev.

[58] Torikai H, Reik A, Liu PQ, Zhou Y, Zhang L, Maiti S (2012). A foundation for universal T-cell based immunotherapy: T cells engineered to express a CD19-specific chimeric-antigen-receptor and eliminate expression of endogenous TCR. Blood.

[59] Jozwik A, Dunlop A, Sanchez K, Benjamin R (2020). Monitoring allogeneic CAR-T cells using flow cytometry. Methods Mol Biology.

[60] Jo S, Das S, Williams A, Chretien AS, Pagliardini T, Le Roy A (2022). Endowing universal CAR T-cell with immune-evasive properties using TALEN-gene editing. Nat Commun.

[61] Tipanee J, Samara-Kuko E, Gevaert T, Chuah MK, VandenDriessche T (2022). Universal allogeneic CAR T cells engineered with sleeping Beauty transposons and CRISPR-CAS9 for cancer immunotherapy. Mol Ther.

[62] Nishimoto KP, Barca T, Azameera A, Makkouk A, Romero JM, Bai L (2022). Allogeneic CD20-targeted γδ T cells exhibit innate and adaptive antitumor activities in preclinical B‐cell lymphoma models. Clin Transl Immunol.

[63] Burnham RE, Zoine JT, Story JY, Garimalla SN, Gibson G, Rae A (2020). Characterization of Donor variability for γδ T cell ex vivo expansion and development of an allogeneic γδ T cell immunotherapy. Front Med.

[64] Rozenbaum M, Meir A, Aharony Y, Itzhaki O, Schachter J, Bank I (2020). Gamma-Delta CAR-T cells Show CAR-Directed and Independent Activity against Leukemia. Front Immunol.

[65] Zhai X, You F, Xiang S, Jiang L, Chen D, Li Y (2021). MUC1-Tn-targeting chimeric antigen receptor-modified Vγ9Vδ2 T cells with enhanced antigen-specific anti-tumor activity. Am J Cancer Res.

[66] Fleischer LC, Becker SA, Ryan RE, Fedanov A, Doering CB, Spencer HT (2020). Non-signaling chimeric Antigen Receptors Enhance Antigen-Directed killing by γδ T cells in contrast to αβ T cells. Mol Ther Oncolytics.

[67] Makkouk A, Yang XC, Barca T, Lucas A, Turkoz M, Wong JTS (2021). Original research: off-the-shelf Vδ1 gamma delta T cells engineered with glypican-3 (GPC-3)-specific chimeric antigen receptor (CAR) and soluble IL-15 display robust antitumor efficacy against hepatocellular carcinoma. J Immunother Cancer.

[68] Polito VA, Cristantielli R, Weber G, Del Bufalo F, Belardinilli T, Arnone CM (2019). Universal Ready-to-Use Immunotherapeutic Approach for the treatment of Cancer: expanded and activated polyclonal γδ memory T cells. Front Immunol.

[69] Li YR, Brown J, Yu Y, Lee D, Zhou K, Dunn ZS (2022). Targeting Immunosuppressive Tumor-Associated Macrophages using innate T cells for enhanced Antitumor Reactivity. Cancers.

[70] Ferry GM, Agbuduwe C, Forrester M, Dunlop S, Chester K, Fisher J (2022). A simple and robust single-step method for CAR-Vδ1 γδT cell expansion and transduction for Cancer Immunotherapy. Front Immunol.

[71] Zeng J, Tang SY, Wang S (2019). Derivation of mimetic γδ T cells endowed with cancer recognition receptors from reprogrammed γδ T cell. PLoS ONE.

[72] Wang Z, McWilliams-Koeppen HP, Reza H, Ostberg JR, Chen W, Wang X (2022). 3D-organoid culture supports differentiation of human CAR + iPSCs into highly functional CAR T cells. Cell.

[73] Iriguchi S, Yasui Y, Kawai Y, Arima S, Kunitomo M, Sato T (2021). A clinically applicable and scalable method to regenerate T-cells from iPSCs for off-the-shelf T-cell immunotherapy. Nat Commun.

[74] Harada S, Ando M, Ando J, Ishii M, Yamaguchi T, Yamazaki S (2022). Dual-antigen targeted iPSC-derived chimeric antigen receptor-T cell therapy for refractory lymphoma. Mol Ther.

[75] Wang B, Iriguchi S, Waseda M, Ueda N, Ueda T, Xu H (2021). Generation of hypoimmunogenic T cells from genetically engineered allogeneic human induced pluripotent stem cells. Nat Biomed Eng.

[76] Ueda T, Kaneko S (2019). In Vitro differentiation of T cell: from CAR-Modified T-iPSC. Methods Mol Biol.

[77] van der Stegen SJC, Lindenbergh PL, Petrovic RM, Xie H, Diop MP, Alexeeva V (2022). Generation of T-cell-receptor-negative CD8αβ-positive CAR T cells from T-cell-derived induced pluripotent stem cells. Nat Biomed Eng.

[78] Boyd N, Cartledge K, Cao H, Evtimov V, Pupovac A, Trounson A (2021). Off-the-Shelf’ Immunotherapy: manufacture of CD8 + T cells derived from hematopoietic stem cells. Cells.

[79] Van Caeneghem Y, De Munter S, Tieppo P, Goetgeluk G, Weening K, Verstichel G (2017). Antigen receptor-redirected T cells derived from hematopoietic precursor cells lack expression of the endogenous TCR/CD3 receptor and exhibit specific antitumor capacities. Oncoimmunol.

[80] Liu DD, Hong WC, Qiu KY, Li XY, Liu Y, Zhu LW (2022). Umbilical cord blood: a promising source for allogeneic CAR-T cells. Front Oncol.

[81] Kim-Hoehamer YI, Riberdy JM, Zheng F, Park JJ, Shang N, Métais JY (2023). Development of a cGMP-compliant process to manufacture donor-derived, CD45RA-depleted memory CD19-CAR T cells. Gene Ther.

[82] Wang X, Naranjo A, Brown CE, Bautista C, Wong CW, Chang WC (2012). Phenotypic and functional attributes of Lentivirus modified CD19-specific human CD8 + central memory T cells manufactured at clinical scale. J Immunother.

[83] Fernández L, Fernández A, Mirones I, Escudero A, Cardoso L, Vela M (2019). GMP-Compliant Manufacturing of NKG2D CAR memory T cells using CliniMACS prodigy. Front Immunol.

[84] Omer B, Castillo PA, Tashiro H, Shum T, Huynh MTA, Cardenas M (2018). Chimeric antigen receptor signaling domains differentially regulate proliferation and native T cell receptor function in virus-specific T cells. Front Med.

[85] Savoldo B, Rooney CM, Di Stasi A, Abken H, Hombach A, Foster AE (2007). Epstein Barr virus–specific cytotoxic T lymphocytes expressing the anti-CD30ζ artificial chimeric T-cell receptor for immunotherapy of Hodgkin disease. Blood.

[86] Ahmed N, Brawley V, Hegde M, Bielamowicz K, Kalra M, Landi D (2017). HER2-specific chimeric antigen receptor–modified virus-specific T cells for progressive glioblastoma: a phase 1 dose-escalation trial. JAMA Oncol.

[87] Morita D, Nishio N, Saito S, Tanaka M, Kawashima N, Okuno Y (2018). Enhanced expression of Anti-CD19 chimeric Antigen receptor in piggyBac Transposon-Engineered T cells. Methods Clin Dev.

[88] Nakazawa Y, Huye LE, Salsman VS, Leen AM, Ahmed N, Rollins L (2011). PiggyBac-mediated Cancer immunotherapy using EBV-specific cytotoxic T-cells expressing HER2-specific chimeric Antigen receptor. Mol Ther.

[89] Cooper LJN, Al-Kadhimi Z, Serrano LM, Pfeiffer T, Olivares S, Castro A (2005). Enhanced antilymphoma efficacy of CD19-redirected influenza MP1-specific CTLs by cotransfer of T cells modified to present influenza MP1. Blood.

[90] Omer B, Cardenas MG, Pfeiffer T, Daum R, Huynh M, Sharma S (2022). A costimulatory CAR improves TCR-based Cancer Immunotherapy. Cancer Immunol Res.

[91] Perna SK, Pagliara D, Mahendravada A, Liu H, Brenner M, Savoldo B (2014). Interleukin-7 mediates selective expansion of tumor-redirected cytotoxic T lymphocytes without enhancement of regulatory T-cell inhibition. Clin Cancer Res.

[92] Magnani CF, Turazzi N, Benedicenti F, Calabria A, Tenderini E, Tettamanti S (2016). Immunotherapy of acute leukemia by chimeric antigen receptor-modified lymphocytes using an improved sleeping Beauty transposon platform. Oncotarget.

[93] Galetto R, Lebuhotel C, Poirot L, Gouble A, Toribio ML, Smith J (2014). Pre-TCRα supports CD3-dependent reactivation and expansion of TCRα-deficient primary human T-cells. Mol Ther Methods Clin Dev.

[94] Stenger D, Stief TA, Kaeuferle T, Willier S, Rataj F, Schober K (2020). Endogenous TCR promotes in vivo persistence of CD19-CAR-T cells compared to a CRISPR/Cas9-mediated TCR knockout CAR. Blood.

[95] Guo Y, Xu B, Wu Z, Bo J, Tong C, Chen D (2021). Mutant B2M-HLA-E and B2M-HLA-G fusion proteins protects universal chimeric antigen receptor-modified T cells from allogeneic NK cell-mediated lysis. Eur J Immunol.

[96] Maalej KM, Merhi M, Inchakalody VP, Mestiri S, Alam M, Maccalli C (2023). CAR-cell therapy in the era of solid tumor treatment: current challenges and emerging therapeutic advances. Mol Cancer.

[97] Yan L, Qu S, Shang J, Shi X, Kang L, Xu N (2021). Sequential CD19 and BCMA-specific CAR T‐cell treatment elicits sustained remission of relapsed and/or refractory myeloma. Cancer Med.

[98] Moradi S, Mahdizadeh H, Šarić T, Kim J, Harati J, Shahsavarani H (2019). Research and therapy with induced pluripotent stem cells (iPSCs): Social, legal, and ethical considerations. Stem Cell Res Ther.

[99] Gattinoni L, Speiser DE, Lichterfeld M, Bonini C (2017). T memory stem cells in health and disease. Nat Med.

[100] Wang X, Popplewell LL, Wagner JR, Naranjo A, Blanchard MS, Mott MR (2016). Phase 1 studies of central memory-derived CD19 CAR T-cell therapy following autologous HSCT in patients with B-cell NHL. Blood.

[101] Biasco L, Scala S, Ricci LB, Dionisio F, Baricordi C, Calabria A (2015). In vivo tracking of T cells in humans unveils decade-long survival and activity of genetically modified T memory stem cells. Sci Transl Med.

[102] Zhou J, Jin L, Wang F, Zhang Y, Liu B, Zhao T (2019). Chimeric antigen receptor T (CAR-T) cells expanded with IL-7/IL-15 mediate superior antitumor effects. Protein Cell.

[103] Rostamian H, Fallah-Mehrjardi K, Khakpoor-Koosheh M, Pawelek JM, Hadjati J, Brown CE (2021). A metabolic switch to memory CAR T cells: implications for cancer treatment. Cancer Lett.

[104] Boulch M, Cazaux M, Cuffel A, Guerin MV, Garcia Z, Alonso R (2023). Tumor-intrinsic sensitivity to the pro-apoptotic effects of IFN-γ is a major determinant of CD4 + CAR T-cell antitumor activity. Nat Cancer.

[105] Wang X, Diamond DJ, Forman SJ, Nakamura R (2021). Development of CMV-CD19 bi-specific CAR T cells with post-infusion in vivo boost using an anti-CMV vaccine. Int J Hematol.

[106] Magnani CF, Gaipa G, Lussana F, Belotti D, Gritti G, Napolitano S (2020). Sleeping beauty–engineered CAR T cells achieve antileukemic activity without severe toxicities. J Clin Invest.

[107] Franceschetti M, Pievani A, Borleri G, Vago L, Fleischhauer K, Golay J (2009). Cytokine-induced killer cells are terminallydifferentiated activated CD8 cytotoxic T-EMRA lymphocytes. Exp Hematol.

[108] Ruella M, Xu J, Barrett DM, Fraietta JA, Reich TJ, Ambrose DE (2018). Induction of resistance to chimeric antigen receptor T cell therapy by transduction of a single leukemic B cell. Nat Med.

[109] Guo C, Ma X, Gao F, Guo Y (2023). Off-target effects in CRISPR/Cas9 gene editing. Front Bioeng Biotechnol.

[110] Tanaka M, Tashiro H, Omer B, Lapteva N, Ando J, Ngo M (2017). Vaccination targeting native receptors to enhance the function and proliferation of chimeric Antigen receptor (CAR)-modified T cells. Clin Cancer Res.

[111] Caruana I, Weber G, Ballard BC, Wood MS, Savoldo B, Dotti G (2015). K562-derived whole-cell vaccine enhances antitumor responses of CAR-redirected virus-specific cytotoxic t lymphocytes in vivo. Clin Cancer Res.

[112] Arcangeli S, Bove C, Mezzanotte C, Camisa B, Falcone L, Manfredi F (2022). CAR T cell manufacturing from naive/stem memory T lymphocytes enhances antitumor responses while curtailing cytokine release syndrome. J Clin Invest.

